# Student's perception of E-learning during COVID-19 pandemic and its positive and negative learning outcomes among medical students: A country-wise study conducted in Pakistan and Iran

**DOI:** 10.1016/j.amsu.2022.104713

**Published:** 2022-09-22

**Authors:** Shahzaib Maqbool, Muhammad Farhan, Hafiz Abu Safian, Iqra Zulqarnain, Hamza Asif, Zara Noor, Mohammad Yavari, Sajeel Saeed, Khawar Abbas, Jawad Basit, Mohammad Ebad Ur Rehman

**Affiliations:** a House Officer, Holy Family Hospital, Rawalpindi, Pakistan; b Rawalpindi Medical University, Rawalpindi, Pakistan; c Quaid-e-Azam Medical College, Bahawalpur, Pakistan; d Islamic Azad University, Tehran, Medical Sciences, Tehran, Iran; e Department of Surgery, Rawalpindi Medical University, Rawalpindi, Pakistan

**Keywords:** COVID-19, E-Learning, Negative outcomes, Positive outcomes, Pandemic

## Abstract

**Background:**

Since the emergence of coronavirus diseases 2019 (COVID-19) not only have social and the economical dimension of life been disturbed but it has also shattered educational activities as well. Due to fear of disease spread educational institutes are forced to implement online educational systems to teach their pupils. This study aims to explore the student's perceptions related to E-learning and their positive and negative outcome among medical students in Pakistan and Iran.

**Method:**

*ology*: This cross-sectional questionnaire-based study was conducted at Rawalpindi Medical University, Rawalpindi, Pakistan, and Islamic Azad University of Medical Sciences Tehran, Iran. This study involved 402 medical students of Rawalpindi Medical University, Rawalpindi, Pakistan (n = 202), and Islamic Azad University of Medical Sciences Tehran, Iran (n = 200) who were actively involved in the online mode of education. A self-administered validated questionnaire was used for data collection. Descriptive statistics and chi-square analysis was used and analysis was done through SPSS V.23. A P-value of 0.05 was taken as significant.

**Results:**

A total of 402 students were enrolled, 202 from Pakistan and 200 from Iran. About 68.2% of the students were acquainted with moderate levels of IT skills. About 75.8% of the students were not showing any previous experience with E-learning. The most common advantage of E-learning was the ability to stay at home. The technical problem was the most common disadvantage in our study. Finally, face-to-face learning in terms of increasing knowledge, skill, and social competence was considered the best mode of learning both by Pakistani and Irani students. Country-wise effectiveness of face-to-face learning in terms of increasing knowledge is statistically significant (p-value = 0.019). Acceptance of E-learning is statistically associated with the country of learning (p-value = 0.020). E-learning was rated as enjoyable by 51.5% of the students.

**Conclusion:**

E-learning has its associated advantages and disadvantages as perceived by medical students but still face-to-face learning is considered the most effective form of learning as responded by medical students.

## Highlights

• The ability to stay at home was the most frequent advantage reported by 64.9% of the Pakistani students as compared to 32% of Irani students.
• Technical problems regarding e-learning were the most frequent disadvantage among Pakistani and Irani students.
• In terms of activity, 5% and 10.9% of students remain extremely inactive and extremely active respectively during traditional face-to-face learning.
• 18.5% and 3.6% of students remain extremely inactive and extremely active respectively during e-learning and the results are statistically significant (p-value<0.00).
• Acceptance of e-learning is statistically associated with country of learning (p-value = 0.020) whereas it is not statistically associated with year of study (p-value = 0.679) and IT skills (p-value = 0.06).

## 1 Introduction

The COVID-19 pandemic is a wave of uncontrolled destruction that has not only shattered the social and economical dimensions of life but has also torn apart the educational facets as well [1]. With the declaration of COVID-19 as a global pandemic, the schools, colleges, and universities of the highly contagious areas of the world were instantaneously closed to mitigate the deleterious effects of COVID-19 [2]. In light of the prevailing pandemic, an abrupt shifting of the educational system was made from face-to-face learning to online methodologies to provide uninterrupted education to the students of the affected countries [3]. In this perspective, various Learning Management Systems (LMS) were launched at various capacities to make the educational system streamlined with a positive future impact on students learning and capabilities under the cover of the COVID-19 pandemic.

Medical education is considered a unique form of education as compared to other educational domains offered at higher institutions of learning [4]. In medical education, students are not only expected to study books but also have to acquire clinical skills as a part of clinical clerkship, particularly during clinical years [4]. In this regard, E-learning was initially thought to compromise the clinical skills of medical students but on the other side, it was a need of time to restrict face-to-face learning to prevent the transmission of SARS-CoV-2 among students and patients. However, a study has also supported the positive impact of E-learning in increasing not only the clinical and basic subject knowledge, but also imparting a beneficial impact in terms of increasing the clinical skills to deal with patients as well [5]. Similar studies conducted worldwide on E-learning have validated the significance and effectiveness of E-learning and its wide-based adoption has also been appreciated by many learners throughout the globe [6,7].

E-learning also known as online education is defined as the learning utilization of electronic technologies to access educational curriculum outside of traditional face-to-face learning in the classroom [8]. Many universities, colleges, and schools are now inclining towards this mode of education delivery worldwide. Before the era of COVID-19, E-learning was not considered the part of formal education delivery system in Pakistan, but with the implementation of a strict lockdown situation due to the increasing number of COVID-19 cases many institutions throughout the country were forced to adopt this mode of education for educating their pupils [9]. Despite having paramount advantages of E-learning in terms of ease of use, better-controlled environment, and overall acceptability by the students, still there are a few limitations of paramount significance that hinder the way of flourishing E-learning mode of education. The most commonly observed disadvantages of E-learning from the perspective of medical students during the COVID-19 pandemic were lack of interaction with teachers and patients, poor connectivity during online classes, a below-par attitude of the students toward online education, and finally lack of acquaintance with both teachers and students towards an online mode of education [10,11]. This particular study was conducted to analyze the experience of medical students towards online education and to explore various advantages and disadvantages that students have to face during online learning so that necessary adaptations for imparting a positive impact while taking online classes in the future could be achieved.

The purpose of this study was to know how medical students of Pakistan and Iran perceived the online mode of education by demonstrating the perceived advantages and disadvantages of E-learning programs during the face of the COVID-19 pandemic. This study also highlighted the positive and negative learning outcomes of online and face-to-face learning during the COVID-19 pandemic.

## 2 Methodology

### 2.1 Study design, period, and setting

This is cross-sectional descriptive questionnaire-based study was conducted from December 2020 to February 2021 at Rawalpindi Medical University, Rawalpindi, Pakistan, and Islamic Azad University of Medical Sciences, Tehran, Iran where medical institutions from both countries were indulged in delivering online education to their students. This study has been conducted following Standards for Reporting Qualitative Research criteria and guidelines [12]. Ethical approval from the institutional review board of Rawalpindi Medical University was taken before the conduction of the study and data collection.

### 2.2 Sampling technique

The convenient sampling technique was followed during the recruitment of the study population. All those students were properly informed about the nature and purpose of the given study and their consent was taken properly. The consent form was part of the response questionnaire and it was marked compulsory to fill the consent before continuing with the remaining response form.

### 2.3 Study population and sample size determination

The sample size calculation was done through Raosoft software. A total population of 3250 with 1750 students from Rawalpindi Medical University and 1500 students from Islamic Azad University of Medical Sciences, was taken as population size and keeping a margin of error of 5%, confidence interval of 95%, with a response distribution of 50%, a sample size of 344 students was calculated but we intentionally took the sample size of 402 students, the reason for oversampling was to account for non-eligibility and non-responders rates and taking our study results more towards generalization.

### 2.4 Inclusion and exclusion criteria

A total of 402 medical students of Rawalpindi Medical University and Islamic Azad University of Medical Sciences were recruited for our study. The students from the first year to final year MBBS from both institutions were included and students of dentistry and allied health sciences were excluded from our study population. All those students who were not involved in taking online classes were also excluded from our study population.

### 2.5 Study questionnaire and data collection technique

The self-administered questionnaire was constructed through a literature search. The questionnaire was comprised of 4 parts. In the first part, demographic details like gender, age, year of study, country of study, previous experience of online education, and information technology skills on a scale of high, moderate, and low levels were assessed. In the second part, the positive and negative learning outcomes of E-learning were assessed in terms of the advantages and disadvantages of online education. Students were provided with 6 sets of options regarding the advantages and disadvantages of online learning and responders were allowed to choose as many options as were true for them. In the third part, the effectiveness of learning objectives like knowledge, clinical skills, and social competence was assessed by comparing two modes of education (online and face-to-face) based on a Likert scale (1 = extremely ineffective, 5 = extremely effective). In the fourth part, the level of acceptance of online learning was assessed by the same Likert scale as above (1 = extremely unenjoyable, 5 = extremely enjoyable). Data collection either from Pakistan or Iran was done through online Google Forms that were sent to the responders through various social media platforms like WhatsApp, Messenger, and data organization and storage were done in a Google Sheet.

### 2.6 Statistical analysis

Statistical analyses were carried out using SPSS V-23.0. Descriptive statistics were applied to determine the frequencies and percentage of distribution of age, gender, country-wise distribution, year of study, IT skills, and previous experience in E-learning. Cross-tabulation and chi-square test was applied to compare the advantages and disadvantages of E-learning in Pakistani and Irani students. The chi-square test was also applied to determine the country-wise effectiveness of E-learning vs face-to-face learning in Pakistani and Irani students. Acceptance of E-learning was determined using descriptive analysis and chi-square analysis. A P-value of less than 0.05 was considered statistically significant.

## 3 Results

### 3.1 Demographic characterizations

Out of 402 students participated in our study, (n = 174, 43.28%) were male and (n = 228, 56.72%) were female. The mean age of students was 21.49 ± 1.79. According to country-wise distribution, 202 students participated from Pakistan and 200 from Iran. Overall, distribution of students according to year of study in increasing order is as follows: second year (n = 6, 15.6%), fourth year (n = 70, 17.5%), first year (n = 73, 18.2%), final year (n = 83,20.5%), third year (n = 113, 28.1%). The majority of the students i.e., (n = 274, 68.2%) have moderate levels of IT skills while only (n = 32, 8%) have a high level of IT skills. Characteristics of the population are summarized in Table 1. Around (n = 38, 18.8%) and (n = 70, 35%) of Pakistani and Irani students respectively have participated in any E-learning before the pandemic and the results are statistically significant (p-value<0.002) according to country-wise crosstabulation.

**Table 1:** Demographic details of the study respondents.

Variables		n (%)
**Gender**	Male	174 (43.28%)
	Female	228 (56.72%)
**Age (years)**	17–20	113 (28.1%)
	21–24	273 (67.9%)
	25–28	16 (4%)
**Country**	Pakistan	202 (50.25%)
	Iran	200 (49.75%)
**Year of Study**	1st	73 (18.2%)
	2nd	63 (15.6%)
	3rd	113 (28.1%)
	4th	70 (17.5%)
	5th	83 (20.5%)
**IT skills**	Low	96 (23.8%)
	Moderate	274 (68.2%)
	High	32 (8%)
**Previous experience in E-learning**	Yes	97 (24.2%)
	No	305 (75.8%)

### 3.2 Positive and negative outcomes of E-learning

In terms of advantages of E-learning, the most frequent responses of students were the ability to stay at home (64.7%), learning at your own pace (53%), access to online material (45.5%) and comfortable surroundings (37.1%) whereas the most frequent disadvantages were technical problems (63.2%), lack of interaction with patients (58.7%), reduced interaction with the teacher (55%) and lack of self-discipline (46%). The ability to stay at home was the most frequent advantage reported by 64.9% of the Pakistani students as compared to 32% of Irani students. While technical problems regarding e-learning were the most frequent disadvantage among Pakistani and Irani students. Country-wise cross-tabulation of advantages and disadvantages is summarized in Table 2.

**Table 2:** Positive and Negative outcomes of E-learning.

Variables	N = 402 (100%)	COUNTRY		P-VALUE
		PAKISTAN	IRAN	
Positive Outcomes of E-learning				
Access to online materials	183 (45.5%)	52.5%	15.5%	0.000
Learning on your own pace	213 (53%)	58%	21.5%	0.014
Ability to stay at home	260 (64.7%)	64.9%	32%	0.884
Classes interactivity	36 (8.9%)	9%	4.5%	0.980
Ability to record a meeting	115 (28.6%)	26.8%	16%	0.340
Comfortable surrounding	149 (37.1%)	35.1%	20.5%	0.322
**Negative Outcomes of E-learning**				
Reduced interaction with the teacher	221 (55%)	58.4%	24%	0.087
Technical problems	254 (63.2%)	66.9%	28%	0.066
Lack of interactions with patients	236 (58.7%)	62.9%	25%	0.033
Poor learning conditions at home	180 (44.8%)	47%	20%	0.248
Lack of self-discipline	185 (46%)	52.5%	16.5%	0.001
Social isolation	142 (35.3%)	35.6%	17.5%	0.912

### 3.3 Comparison between E-learning and face-to-face learning

**Fig. 1:**
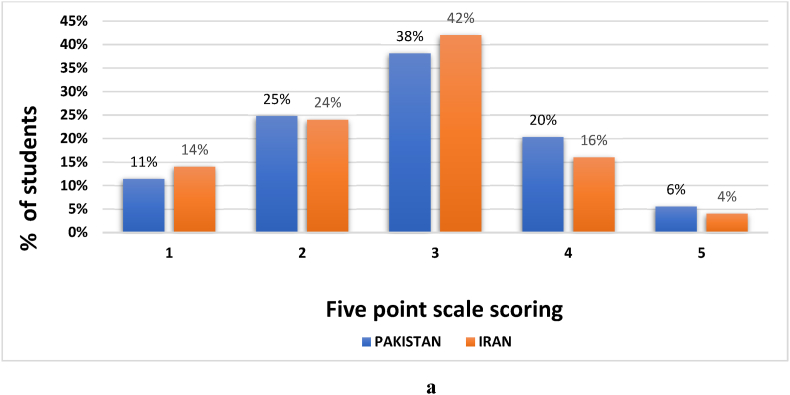

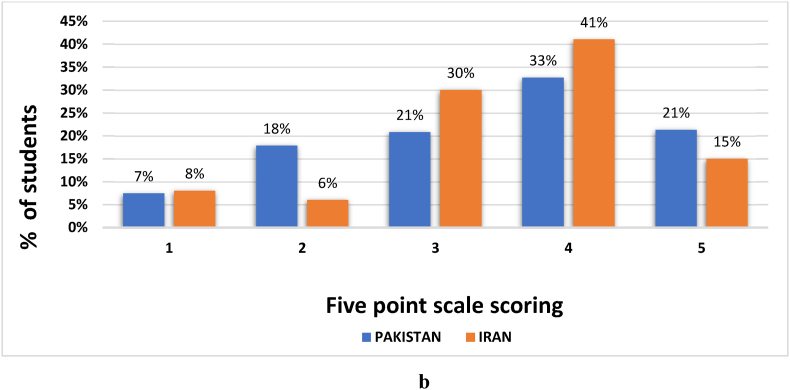
Showing the effectiveness of E-learning (Fig. 1a) and face-to-face (Fig. 1b) learning in terms of increasing knowledge based on the Likert scale (1 = extremely ineffective, 5 = extremely effective) among medical students of Pakistan and Iran.

**Fig. 2:**
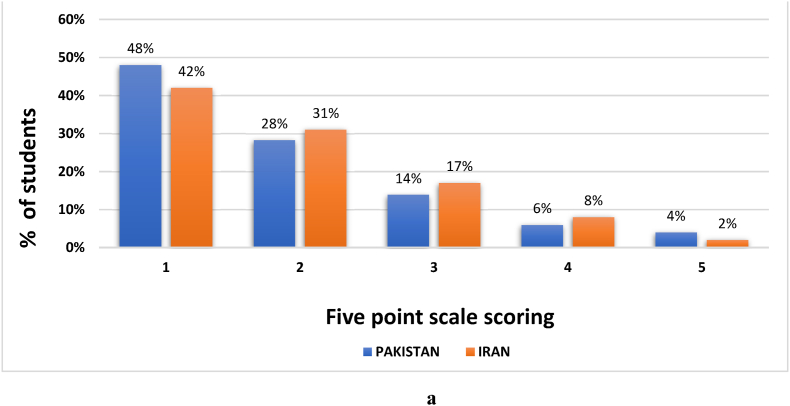

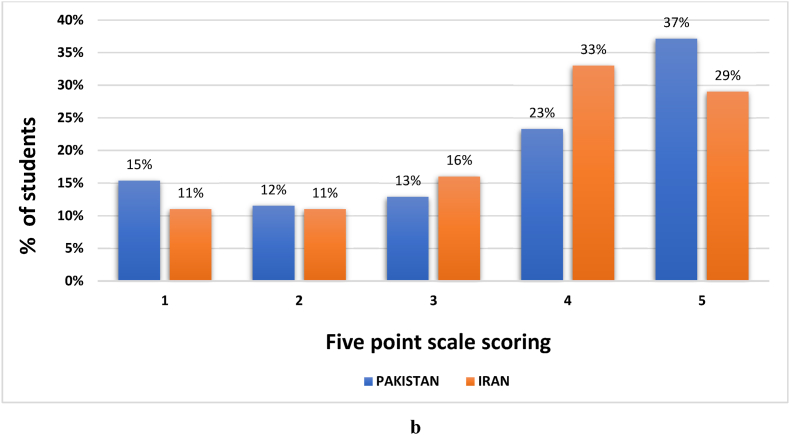
Showing the effectiveness of E-learning (Fig. 2a) and face-to-face (Fig. 2b) learning in terms of increasing clinical skills based on the Likert scale (1 = extremely ineffective, 5 = extremely effective) among medical students of Pakistan and Iran.

**Fig. 3:**
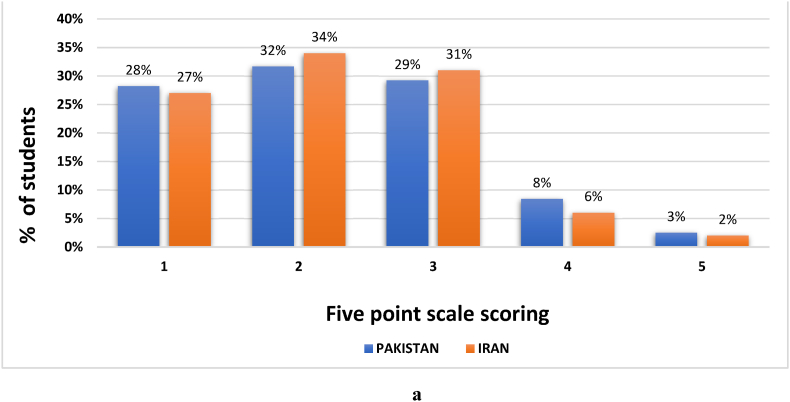

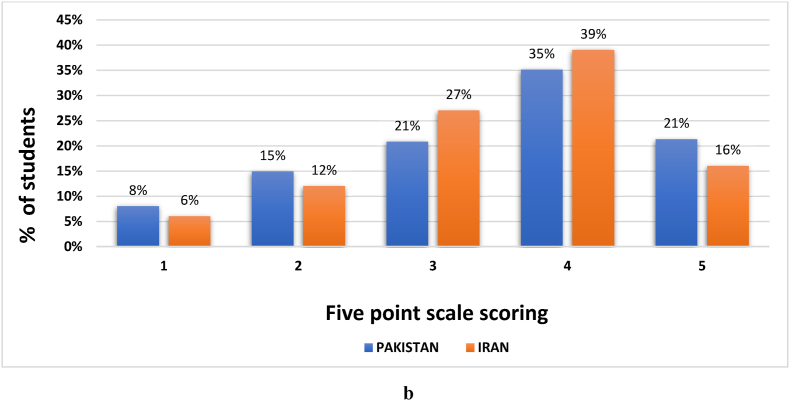
Showing the effectiveness of E-learning (Fig. 3a) and face-to-face learning (Fig. 3b) in terms of increasing social competence based on the Likert scale (1 = extremely ineffective, 5 = extremely effective) among medical students of Pakistan and Iran.

In terms of activity, 5% and 10.9% of students remain extremely inactive and extremely active respectively during traditional face-to-face learning while 18.5% and 3.6% of students remain extremely inactive and extremely active respectively during E-learning and the results are statistically significant (p-value<0.00). Country-wise effectiveness of E-learning in terms of increasing knowledge, clinical skills, and social competencies is illustrated in Fig. 1, Fig. 2, Fig. 3 respectively. Whereas country-wise effectiveness of face-to-face learning in terms of increasing knowledge, clinical skills, and social competencies is illustrated in Fig. 1, Fig. 2, Fig. 3 respectively. Country-wise effectiveness of face-to-face learning in terms of increasing knowledge is statistically significant (p-value<0.019).

### 3.4 Acceptance of E-learning among medical students

**Fig. 4:**
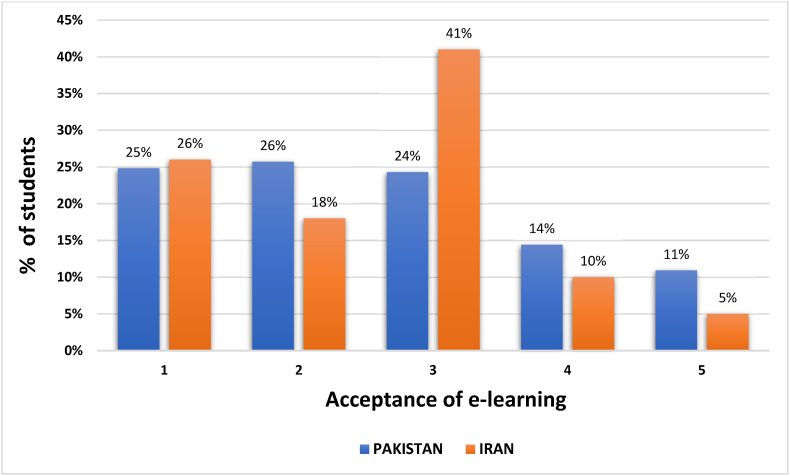
Showing the country-wise acceptance of E-learning based on the Likert scale (1 = extremely unenjoyable, 5 = extremely enjoyable) among medical students of Pakistan and Iran.

Out of 402 students, (n = 207, 51.5%) found E-learning enjoyable and of these, (n = 36, 9%) found it extremely enjoyable, (n = 52, 12.9%) found it very enjoyable, and (n = 119, 29.6%) found it somewhat enjoyable. Total (n = 195, 48.5%) students did not enjoy e-learning and of these (n = 101, 25.1%) found it extremely un-enjoyable and (n = 94, 23.4%) found it very un-enjoyable. Acceptance of e-learning is statistically associated with country of learning (p-value = 0.020) whereas it is not statistically associated with year of study (p-value = 0.679) and IT skills (p-value = 0.06). Country-wise acceptance of e-learning is summarized in Fig. 4.

## 5 Discussion

This comparative study aims to examine the student's perception of online learning and its positive and negative learning outcomes among medical students during the COVID-19 pandemic among medical students of Rawalpindi medical university, Pakistan, and Islamic Azad University of Medical Sciences, Iran.

Out of the total, 402 students enrolled in our study. About (n = 202, 50.25%) of the students were from Pakistan and almost (n = 200, 49.75%) students were from Iran. Most of the students (n = 274, 68.2%) enrolled in this study were having moderate levels of IT skills, this is in contrast to the study conducted on Polish medical students in Poland where most of the students (n = 451, 56%) were acquainted with high levels of IT skills [13]. In this study majority of the student (n = 305, 75.8%) had not experienced an online mode of education before the COVID-19 pandemic which is contrary to a study conducted among medical students of North Jordan where the majority of the students 73.6% had already experienced the online education before COVID-19 pandemic [14]. The possible reason for the lack of IT skills and previous experience of online learning among students of the current study could be the lack of emergence of education and learning technologies and the lack of their utilization in Pakistan and Iran as compared to countries like Poland and Jordan. Another reason could be that in the majority of the countries in the central and middle East, traditional face-to-face learning is considered the preferred mode of education with less preponderance towards online classes contributing to a lack of skills in information technology [9].

The positive and negative outcomes of E-learning were described in terms of advantages and disadvantages perceived by medical students while taking online classes. The most commonly observed advantage of E-learning perceived by about (n = 260, 64.7%) of the medical students was their ability to stay at home. Similarly, concerning country-wise distribution ability to stay at home was the common advantage perceived by the students of both Pakistan and Iran. These findings are in concordance with a study conducted on Polish medical students of Poland who also showed the same advantage of the ability to stay at home [13]. The possible reason for this commonly observed advantage may be the reduction in the cost of accommodation, transportation, and a comfortable and flexible environment at home [15]. Similarly, the other commonly observed advantage was learning at your own pace followed by access to online study materials, comfortable surroundings, ability to record a meeting. Finally, class interactivity was the least commonly observed advantage we assessed in our study. In country-wise distribution ability to stay at home was the main advantage observed among both Pakistani and Irani students commonly.

Similarly, the most common disadvantage that was observed in our study was an inability to handle the technical problems faced during e-learning. The most common technical problem faced by the students was poor internet connections during online classes. This finding was in concordance with other studies conducted to assess the disadvantages of online learning which were also validating the problem of internet connectivity during online classes [14]. Many studies have also reported that excessive use of internet networks in almost every field of life during the COVID-19 pandemic was the main cause of slow internet connections [16]. In the same vein, a study conducted in Kerala was also reporting the problem of internet connectivity and associated technical issues among 43.7% of the students who were taking online classes [17]. Similarly, other barriers hindering the successful delivery of online education were poor infrastructure and a lack of skilled staff for delivering online lectures [18]. The other disadvantages were lack of interaction with patients and teachers as consistent with other studies conducted to assess the medical student's perceptions of E-learning during the COVID-19 pandemic [19,20], followed by lack of self-discipline, poor learning conditions at home, and social isolation. The same pattern of disadvantages had been observed when country-wise distributions of disadvantages were cross-tabulated among students of Pakistan and Iran. These findings were consistence with a study conducted in Poland [13]. Many institutions are now adapting to overcome these disadvantages, but the learning curve to minimize these negative outcomes is still on the higher side.

The third part of our study is to evaluate the effectiveness of E-learning vs face-to-face learning among medical students of Rawalpindi Medical University and Islamic Azad University of Medical Sciences. Three variables of interest such as knowledge, clinical skills, and social competence were evaluated and their levels of effectiveness were compared between two comparison groups of E-learning and face-to-face learning. Most of the students opted that face-to-face learning is considered more effective than E-learning in increasing their knowledge, clinical skills, and social competence as shown in Fig. 1, Fig. 2, Fig. 3. These findings are consistent with other studies conducted to evaluate the levels of effectiveness between E-learning and face-to-face learning [21,22]. Though the students from both Pakistan and Iran were favoring face-to-face learning but the students from Islamic Azad University of Medical Sciences, Iran were showing more preponderance towards face-to-face learning for increasing knowledge and clinical skills; however, students from Pakistan were showing that face-to-face learning is more effective in increasing social competence followed by knowledge and clinical skills. In the same vein, some studies have also depicted that E-learning is more effective in increasing knowledge as compared to face-to-face learning [13]. The most common barriers to rendering E-learning being less effective are lack of proper infrastructure, poor attitude of the students, poor internet connectivity, lack of experience by the instructors, and inability to deliver the clinical demonstration through the platform of E-learning [23]. Because online learning was not considered an effective platform for teaching medical students the level of acceptance for E-learning was just 51.5% as compared to 73% observed in Polish medical students in Poland and the possible reason could be the lack of resources and affordability during lockdown conditions in Pakistan and Iran [13].

### 5.1 Limitations and recommendations

The possible limitation of our study is a low sample size which is why study results could not be generalized. The other limitation is a study design that could also have limited objectivity and more importantly, institutional bias can occur due to data collection from two universities. We recommend that other studies related to online learning should be carried out so that the insight of students in terms of positive and negative impact could be explored and necessary steps to mitigate the negative learning outcome could be undertaken.

## 6 Conclusion

In this study, we evaluated the student's perception of E-learning and its associated advantages and disadvantages in terms of learning outcomes were studied. The students from Pakistan and Iran were included in our study. The majority of the students in our study were showing moderate levels of previous IT skills and the majority of the students were lacking previous experience E-leaning in our study. Regarding advantages, the ability to stay at home was the most commonly observed positive outcome of E-learning among medical students of Pakistan and Iran followed by learning at your own pace and access to online materials. Similarly, the technical problem was the most common disadvantage observed in our study followed by a lack of interaction with the patients and teachers. While comparing E-learning and face-to-face learning among students of Pakistan and Iran, face-to-face learning was considered the most effective way of learning to increase knowledge, clinical skills, and social competence. The overall acceptability of E-learning was observed in half of the sample population where half of the students were enjoying the E-learning platform of education delivery and the rest of the students were not enjoying online classes.

## Ethical approval

Ethical approval not required.

## Sources of funding for your research

No funding required for the study.

## Author contribution

Shahzaib Maqbool, Muhammad Farhan, and Hafiz Abu Safian: Conception, design, write-up, critical review and approval of the final version. Iqra Zulqarnain, Hamza Asif, Zara Noor, and Mohammad Yavari: Write-up, critical review and approval of the final version.Sajeel Saeed, Khawar Abbas, Jawad Basit and Mohammad Ebad ur Rehman: Editing, critical review and approval of the final version.

## Registration of research studies

1. Name of the registry: Not applicable
2. Unique Identifying number or registration ID: Not applicable
3. Hyperlink to your specific registration (must be publicly accessible and will be checked): Not applicable

## Guarantor

Shahzaib Maqbool, House Officer, Holy Family Hospital, Rawalpindi, Pakistan.

Muhammad Farhan, MBBS, Rawalpindi Medical University, Rawalpindi, Pakistan.

Hafiz Abu Safian, House Officer, Holy Family Hospital, Rawalpindi, Pakistan.

## Consent

Not required.

## Provenance and peer review

Not commissioned, externally peer-reviewed.

## Declaration of competing interest

All authors declared no conflict of interest.
