# Supplementary material for: Student's perception of E-learning during COVID-19 pandemic and its positive and negative learning outcomes among medical students: A country-wise study conducted in Pakistan and Iran
Source: Ann Med Surg (Lond). 2022 Sep 22;82:104713. doi: 10.1016/j.amsu.2022.104713 (PMC9494861; doi:10.1016/j.amsu.2022.104713)
Supplement: Multimedia component 1 [file mmc1.docx]

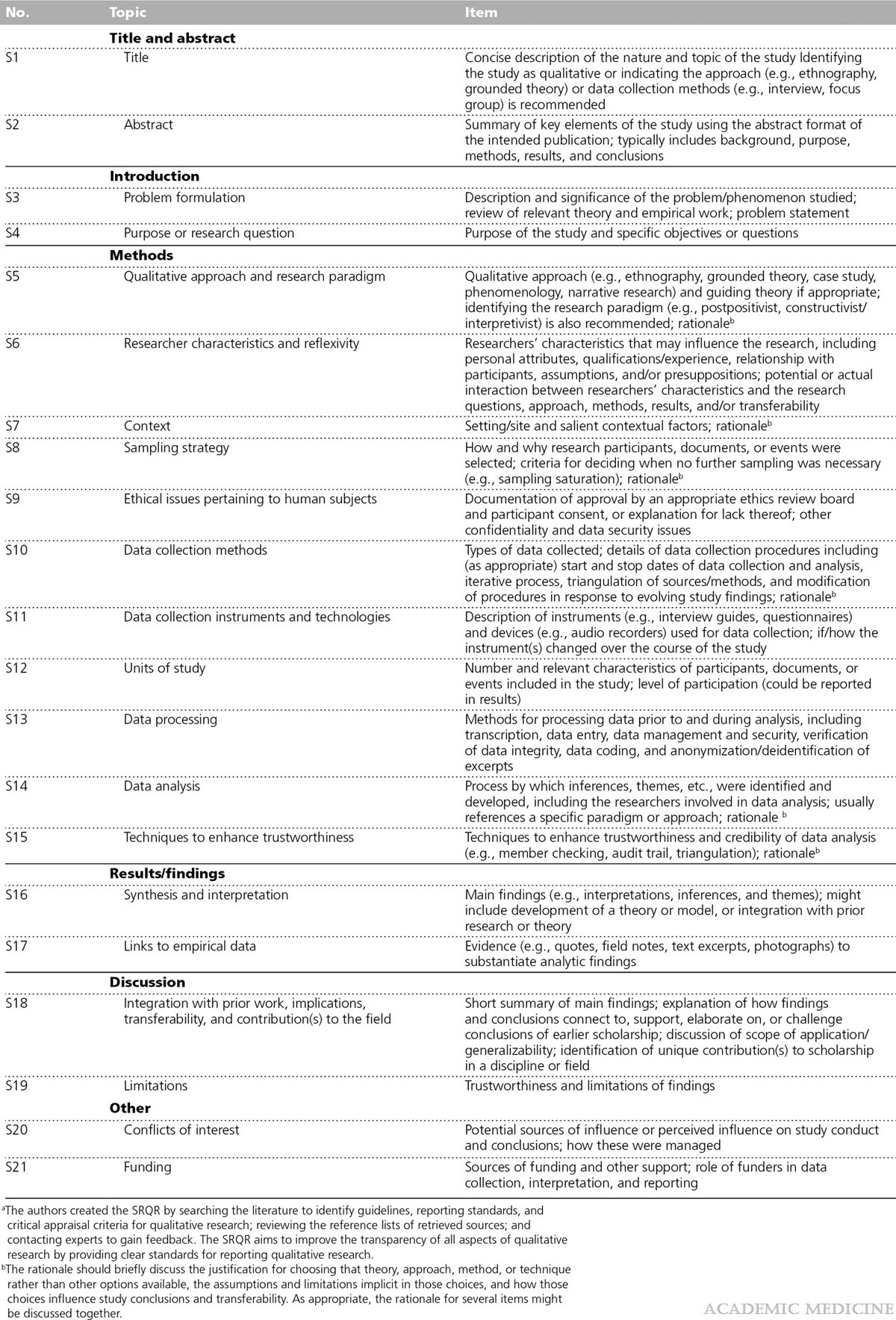


S 17 is not applicable. Present on page no 6 to 12 on manuscript file.

S5, S6, S7 and S 15 are not applicable. Remaining all components of method section are present on page no 4 of manuscript file.

Present on page no 17 of manuscript file.

Present on page no 13 to 15 on manuscript file.

Present on page no 2 and 3 of manuscript file.

Present on page no 1 of manuscript file.

Present on Title page; which is a separate file.

**Standards for Reporting Qualitative Research Checklist**
